# Dogbury down on the Rubbers

**Published:** 1888-01

**Authors:** 


### DOGBURY DOWN ON THE RUBBERS.

Dr. James Walsh was recently tried in Special Sessions for practicing medicine at 119 East Eighty-fourth street without a diploma. The complainant was W. A. Perrington, counsel of the Medical Society. Henry Levien a spotter testified that he called on the alleged doctor on Dec. 3, and asked to be treated for rheumatism. The doctor rubbed vigorously for some time, and then gave him a bottle of medicine. The defendant said he had a sign upon the door of his residence with the word doctor upon it, but admitted that he was not a practicing physician, but only a rheumatic doctor. He was a rubber. The doctor remembered that Mr. Levien called on him and he rubbed him for the rheumatism, and gave him a bottle of sugar-house molasses, and told him to add some lemon skin to it, and take some before retiring at night. He took $2 for his services. The Court found the doctor guilty, and imposed a fine of $100, which he said he could not pay.

Had the doctor been convicted of theft or some such little matter, it is quite likely the discriminating Court might have been induced to suspend judgment, but for the heinous crime of releiving a pretended rheumatic *spotter* of the Medical Society, with a bottle of molasses thrown in, mercy would be quite out of place. "One hundred dollars fine," is easily said, but for a poor man to pay, quite out of the question. The sneak trade seems to pay well.

